# The use of minimal topological differences to inspire the design of novel tetrahydroisoquinoline analogues with antimalarial activity

**DOI:** 10.1016/j.heliyon.2021.e07032

**Published:** 2021-05-21

**Authors:** Joelleinsert Ngo Hanna, Vincent de Paul N. Nziko, Fidele Ntie-Kang, James A. Mbah, Flavien A.A. Toze

**Affiliations:** a Department of Chemistry, Faculty of Science, University of Buea, P. O. Box 63, Buea, Cameroon; b Department of Chemistry, Faculty of Science, University of Douala, P. O. Box 24157, Douala, Cameroon; c Department of Chemistry and Biochemistry, Hampton University, Hampton, Virginia, USA; d Department of Pharmaceutical Chemistry, Martin-Luther University of Halle-Wittenberg, Kurt-Mothes-Str. 3, 06120, Halle (Saale), Germany; e Institute of Botany, Technical University University Dresden, Zellescher Weg 20b, 01062, Dresden, Germany

**Keywords:** Gaussian 09W, *In silico* design, Malaria, *Plasmodium falciparum*, QSAR, Screening

## Abstract

A quantitative structure-activity relationship (QSAR) study was conducted using nineteen previously synthesized, and tested 1-aryl-6-hydroxy-1,2,3,4-tetrahydroisoquinolines with proven *in vitro* activities against *Plasmodium falciparum*. In order to computationally design and screen potent antimalarial agents, these compounds with known biological activity ranging from 0.697 to 35.978 μM were geometry optimized at the B3LYP/6-311 + G(d,p) level of theory, using the Gaussian 09W software. To calculate the topological differences, the series of the nineteen compounds was superimposed and a hypermolecule obtained with $\underline{\text{s}}$ = 17 and 20 vertices. Other molecular descriptors were considered in order to build a highly predictive QSAR model. These include the minimal topological differences (MTD), LogP, two dimensional polarity surface area (TDPSA), dipole moment (μ), chemical hardness (η), electrophilicity (ω), potential energy (E_p_), electrostatic energy (E_ele_) and number of rotatable bonds (NRB). By using a training set composed of 15 randomly selected compounds from this series, several QSAR equations were derived. The QSAR equations obtained were then used to attempt to predict the IC_50_ values of 4 remaining compounds in a test (or validation) set. Ten analogues were proposed by a fragment search of a fragment library containing the pharmacophore model of the active compounds contained in the training set. The most active proposed analogue showed a predicted activity within the lower micromolar range.

## 1 Introduction

Malaria is a parasitic disease that causes death and economic loss in about half the population of the world (Bloland, 2001). Malaria caused by *Plasmodium falciparum* is transmitted by female anopheline mosquitoes (Bloland, 2001). According to the World Health Organization (WHO), there were 229 million fatal cases reported in the year 2019 (WHO, 2020). Vector control measures and chemoprophylaxis are among the attempts made to control the disease, but these have had limited success (World Health Organization, 2010). The most effective current method of controlling malaria is by the administration of antimalarial drugs to sick patients (Patel et al., 2003; Vangapandu et al., 2007). Some of the most effective available antimalarial drugs are quinine-based and artemisinin derivatives often used in combination therapy (Diallo et al., 2020). Malaria control has, however, been faced by resistance of the mosquito vector to insecticides. Moreover, new strains of *Plasmodium falciparum,* the most dangerous malaria parasite to humans have also emerged that do not respond to known antimalarials (Ridney, 2002), requiring the need for new antimalarial drugs, including those from natural-product-like scaffolds (Onguéné et al., 2013; Ntie-Kang et al., 2014; Bekono et al., 2020).

Despite lots of efforts that have been made towards the discovery of an effective vaccine against malaria, none has yet been found. With the increased spread of malaria in developing countries, efforts towards the development of new synthetic antimalarial drugs have regained importance. This has also partly been motivated by the increased resistance of vectors to commonly used drugs and insecticides (Menard and Dondorp, 2017). This calls for interest in the search for new antimalarial drugs that target aspects of the malaria parasite genome that are not targeted by antimalarials already in use. Such efforts have been encouraged by the sequencing and cloning of *P. falciparum* kinases, which are protein drug targets that are homologous to their mammalian cyclin-dependent kinase (CDK) counterparts (Carvalho et al., 2013).

CDKs are kinases that control progression in cell cycle within the parasite and have been reported to be potential targets for drug development against malaria caused by *P. falciparum.* The CDKs like *PfPK5* and *Pfmrk* have high sequence and structural similarity when compared to their mammalian homologous proteins, e.g. CDK1 (60% identical) and CDK7 (46% identical) have become attractive targets for novel antimalarial agents (Woodard et al., 2003). *Pfmrk* is a well-characterized CDK protein kinase from *P. falciparum* and has shown significant homology with the human CDK7 (Peng et al., 2005). Oxindoles and indoles, β-carbolines and isoquinolines have been found to be CDK inhibitors.

**Figure 1:**
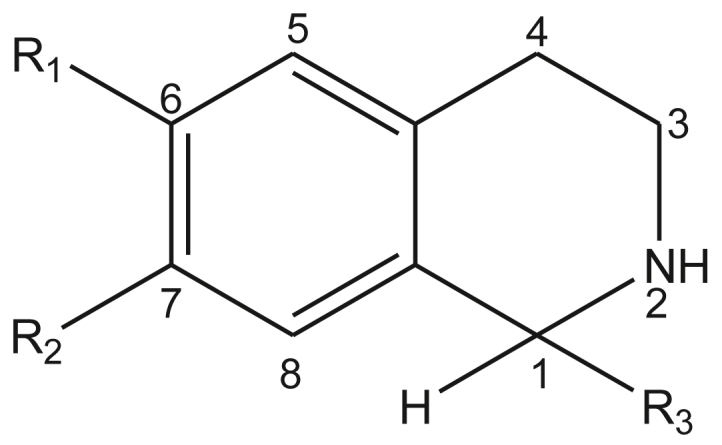
Chemical structure of compounds under study.

In the search for novel antimalarial drugs, scientists have investigated the biosynthesis and identification of known and new tetrahydroisoquinolines. Tetrahydroisoquinoline (Figure 1) is a substructure of the naphthylisoquinoline dioncophylline which has shown antimalarial activity (Bringmann et al., 2008). Tetrahydroisoquinoline, also called 1,2,3,4-tetrahydroisoquinoline (TIQ), is the common core structure of many drugs and many alkaloids isolated from natural sources. Many compounds having this substructure have exhibited diverse biological activities, including antibacterial, antifungal, antimicrobial, anti-human immunodeficiency virus (HIV), antimalarial, antileishmanial, antitumor, antitubercular, antitrypanosomal and cardiovascular activities (Bringmann et al., 2008; Fayez et al., 2018; Kumar et al., 2006, 2010; Li et al., 2017; Moyo et al., 2020).

In this paper, we report the computer-assisted design of a virtual library of tetrahydroisoquinoline analogues, followed by the *in silico* screening by use of quantitative structure-activity relationship (QSAR) methods. The chemical space surrounding the tetrahydroisoquinoline scaffold was studied by substitution at position R_3_ by small and large groups or fragments with hydrophobic characteristics, with the goal of improving the antimalarial activity. Other portions of the scaffold were also modified to improve antimalarial activity. This resulted in a small but highly focused subset of tetrahydroisoquinoline analogues, derived combinatorially, and containing antimalarial *in silico* hits with better activity than the initial set of compounds. In addition, the designed analogues displayed predicted favorable ADME properties.

## 2 Materials and methods

### 2.1 Computer hardware

All the computations were carried out on a desktop work option running on four processors on both Windows and Linux systems. The computers used are Pentium ® Dual core CPU, E5400@2.70GHz, with 3.46 GB of RAM, and the system is Microsoft XP Professional version 2002.

### 2.2 Compound dataset

The synthesis of the training and test set compounds (shown in Table 1) has been previously published (Ngo Hanna et al., 2014), and so far as we know no work has been done towards a QSAR study of antimalarial activities on the tetrahydroisoquinolines.

**Table 1:** Training and test set used in the QSAR model of anti-malarial agents.

Training				
No.	R_1_	R_2_	R_3_	IC_50_ (μM)
1	OH	H	4-chlorophenyl	0.697
2	OH	H	3,4-dichlorophenyl	1.343
3	OH	H	3-chlorophenyl	1.336
4	OH	H	3-methoxyphenyl	2.530
5	OH	H	2,3-dimethoxyphenyl	3.126
6	OH	H	2,5-dimethoxyphenyl	4.276
7	OH	H	4-bromophenyl	1.581
8	OH	H	α,α,α-trifloromethylphenyl	0.760
9	OH	H	biphenyl	3.006
10	OH	H	2-florophenyl	9.495
11	OH	H	4-florophenyl	2.446
12	OH	H	4-chloro-3-nitrophenyl	6.727
13	OH	H	5-bromo-2-methoxyphenyl	2.226
14	OH	H	2-hydroxy-5-nitrophenyl	35.978
15	OH	H	4-methylphenyl	3.955
Test				
16	OH	H	phenyl	2.304
17	OH	H	3-nitrophenyl	1.284
18	OH	H	3-bromophenyl	3.550
19	OH	H	3-florophenyl	6.787

### 2.3 Geometry optimization

Geometry optimizations of the compounds were carried out by implementing the density functional theory (DFT) because this method offers a good compromise of saving computational time while properly describing the electronic correlation, hence physicochemical properties of molecules. Moreover DFT is commonly better than other quantum mechanical methods like the Hartree-Fock (HF) which does not take the electron spin into account. In this work, we employed the B3LYP, which is a hybrid functional variant of the DFT. The B3LYP employs the Becke's three parameter exact exchange functional (B3) (Becke, 1988), and combines it with the non-local gradient corrected correlation functional proposed by Lee, Yang and Parr (LYP) (Lee et al., 1988). Using the Gaussian 09W software (Gaussian, Inc., 2009a, 2009b), the selected basis set was the 6-311+G (d,p), which was used as the wave function. This is a 6–311 split-valence triple zeta Gaussian basis set developed by the Pople research team (Curtiss et al., 1992; Ditchfield et al., 1971; Francl et al., 1982; Hariharan and Pople, 1974; Hehre et al., 1972; Wiberg, 2004), supplemented by a set of *d* and *p* polarization functions (Frisch et al., 1984). The *d* polarization functions are applied on the heavy atoms while the *p* polarization functions are for the hydrogen atoms. These are further supplemented by a set of single diffuse *s* functions (Clark et al., 1983) on both heavy atoms. The geometry was fully optimized with no symmetry constraints up to convergence. The convergence criterion was set as the largest nuclear gradient component = 10^−6^ a.u./Bohr and the change in total energy <10^−7^ a.u.

### 2.4 QSAR studies

A QSAR model for the molecules in the training set was built using molecular descriptors, including those available in the Molecular Operating Environment (MOE 2007.09) software package distributed by the Chemical Computing Group (CCG Inc., 2010). Such descriptors included the potential energy, the electrostatic energy and number of rotatable bonds. Specific descriptors such as molar refractivity (MR) were computed from ACD-Lab software (Advanced Chemistry Development, Inc., 2010); logarithm of the *n*-octanol/water partition coefficients (Xlogp) were computed from the BROOD software (OpenEye Scientific Software, Inc., 2006). The dipole moment of the molecules were obtained from Gaussian 09 (Gaussian, Inc., 2009a, 2009b). The chemical hardness (*η*) and electrophilicity (*ω*) were calculated from the lowest unoccupied molecular orbital (LUMO) energies and highest occupied molecular orbital (HOMO) energies values obtained from Gaussian 09W, according to the following formulae (Eqs. (1) and (2), respectively). The chemical potential is also defined from the HOMO and LUMO orbital energies according to Eq. (3). The minimal topological difference (*MTD*_*i*_) value of each molecule “*i*” with respect to the receptor was calculated for each molecule from the hypermolecule obtained from the superposition of all the molecules. This was computed by using the formula shown in Eq. (4) (Duda-Seiman et al., 2006).

(1) $$\text{Chemical}\;\text{hardness,}\;\eta =\frac{E_{LUMO}-E_{HOMO}}{2}$$

(2) $$\text{Electrophilicity,}\;\omega =\frac{\mu^{2}}{2\eta },$$

(3) $$\text{where}\;\text{μ,}\;\text{the}\;\text{chemical}\;\text{potential}\;\text{is}\;\text{defined}\;\text{as}\;\text{μ}=\frac{E_{LUMO}+E_{HOMO}}{2}$$

When studying the quantitative structure-activity relationships (QSAR) of drugs, the relative potency of the various analogues is considered to be determined by several physicochemical properties. To analyze these relationships, both the calculation of the best least square equation for all data and evaluation of statistical significance of the contribution of each individual predictor variable is carried out. Regression analysis establishes and quantifies the dependence of relative potency on molecular parameters or descriptors. Multiple regressions are done only by the computer. The validity of the QSAR model results is highly dependent on how representative are the training series and the biological data. If the structural variations within the training series are too narrow the predictive power of the resulting model is limited. The result is therefore verified by applying leave-one-out techniques. In this technique, groups of *n* compounds are alternatively left out of discriminant analysis and then classified with the aid of the discriminant function obtained from the remaining compounds. The result can be trusted only if the model remains stable during such procedures.

The inhibitory concentration (*IC*_50_) for evaluating the antimalarial activities tetrahydroisoquinoline analogues was taken from our previous work (Ngo Hanna et al., 2014) (Table 1). The inhibitory concentration (expressed as IC_50_ values in μM) values were converted to the logarithmic forms (p*IC*_50_) with the goal of establishing models that correlate the experimental activities with the calculated molecular descriptors for the 15 training set molecules. The partial-least-squares (PLS) regression method (Cramer et al., 1988) was used to correlate the value of molecular descriptors calculated with the experimental p*IC*_*50*_ (- log *IC*_50_) values. The “leave-one-out” cross validation procedure (Cramer et al., 1988) was repeated for the training set molecules with the aim of searching the highest squared correlation coefficient (*R*^*2*^ values) and the highest number of principal components (PCs). The PLS method was used to fit the QSAR models generated. However, the goal was not to generate just one QSAR model. Our approach consisted in building several QSAR models and choosing among the most predictive ones. The linear regression analysis was carried out using the QuaSAR module of MOE (Chemical Computing Group Inc., 2010). The QSAR equation obtained from the 15 molecules in the training set was verified by applying it to correctly to predict the activities of 4 analogues with known $IC_{50}^{exp}$ values that were left in the validation or test set.

### 2.5 Minimal topological differences (MTD)

The parameter that takes into account the steric properties of whole chemical structures is the MTD concept (earlier referred to as the minimal steric difference). According to the MTD concept, the affinity of a drug towards a given receptor decreases linearly with the non-overlapping volume of the molecule and the receptor cavity. The hypermolecule approach assumes that:

• all the molecules bind to the same binding pocket, and
• the free-ligand minimum-energy conformation is the same as the bound ligand.

**Figure 2:**
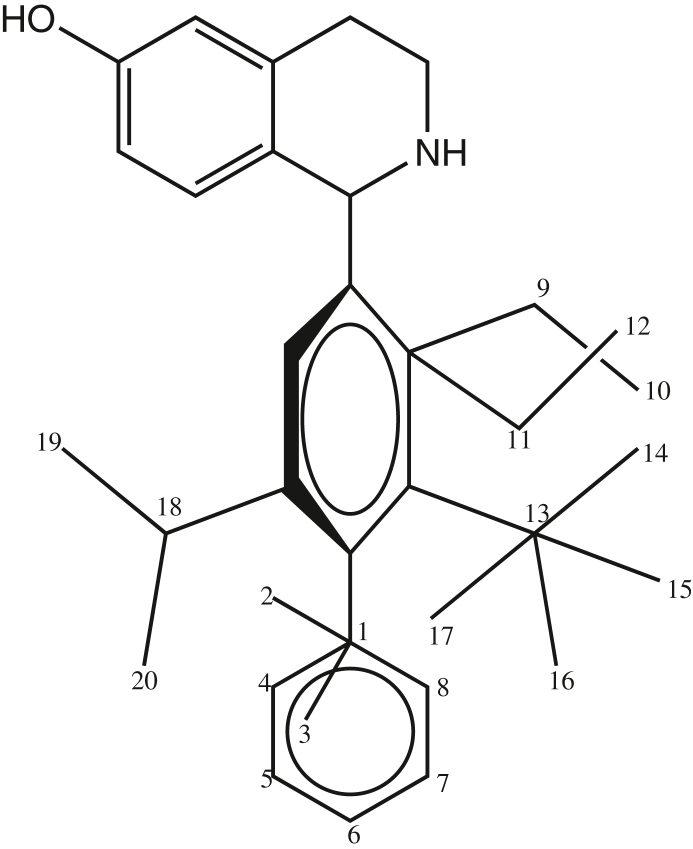
Hypermolecule obtained by superposition of the minimum energy conformations of 19 tetrahydroisoquinoline analogues.

Since the shape and size of the receptor cavity is not known, the volume cannot be calculated directly. Therefore an indirect method was developed in which a “*receptor map*” derived from the structures within the sample compounds considered is used instead of the receptor structure. The non-overlapping volume for a given molecule is then approximated by the MTD *value* which is the number of non-overlapping atoms (hydrogens being neglected) between this molecule and that part of the receptor map which represents the receptor cavity (Figure 2). The principal steps in calculating the MTD are:

1. The structures of all molecules considered are superimposed to give an artificial hypermolecule, H, representing a topological network with the atoms as vertices.
2. Each molecule is superimposed over H. As a result a vector of *k* logical parameters (*k* = number of vertices in H), *x*_*j*_ (*j* = 1, …, *k*), is obtained which describes its structure. For the *i*-th molecule, *x*_*j*_ is defined as: *x*_*ij*_ = 1, if the *j*-th vertex in H is occupied by an atom of the compound *i*; and *x*_*ij*_ = 0, if not.

3. An initial guess is made as to which vertices of the supermolecule H fall into the receptor cavity, the cavity wall or outside the receptor binding site by assigning a ternary parameter $\varepsilon_{j}$ to each vertex: $\varepsilon_{j}$ = -1, if vertex *j* belongs to the space of the receptor cavity; $\varepsilon_{j}$ = 0, if vertex *j* is outside; and $\varepsilon_{j}$ = +1, if vertex *j* belongs to the wall of the cavity.

**Figure 3:**
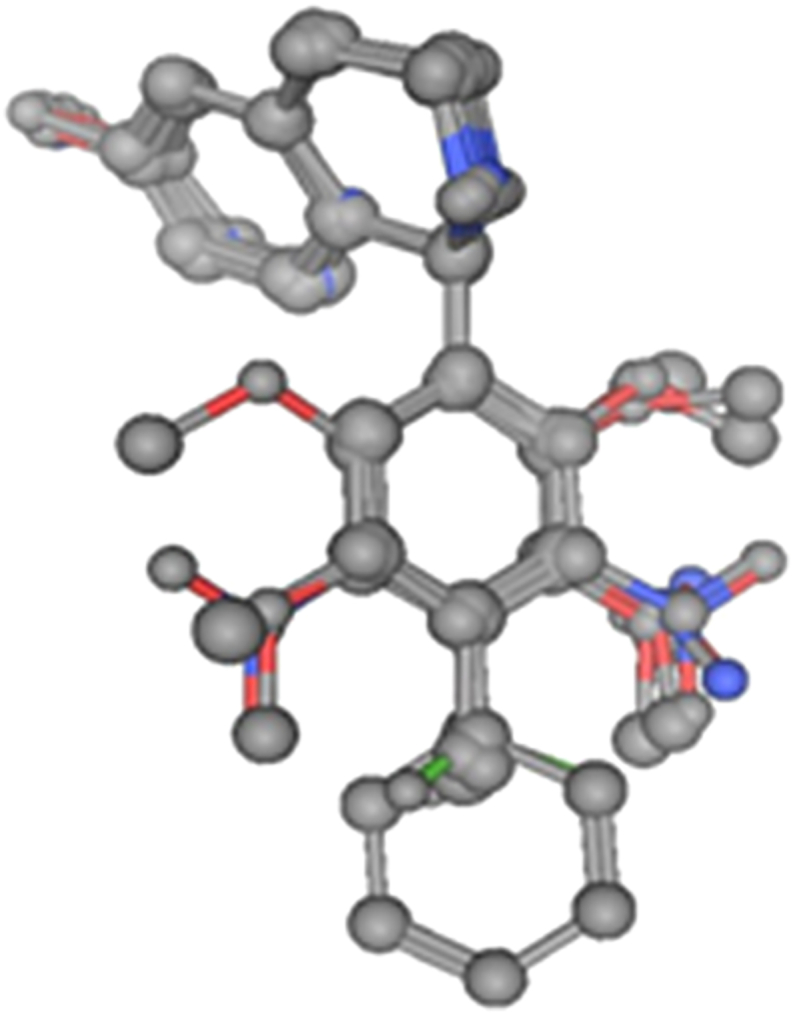
Superposition of the minimal energy conformations of the 19 tetrahydroisoquinoline analogues using the MOPAC method from MOE software (Chemical Computing Group Inc, 2010).

**Figure 4:**
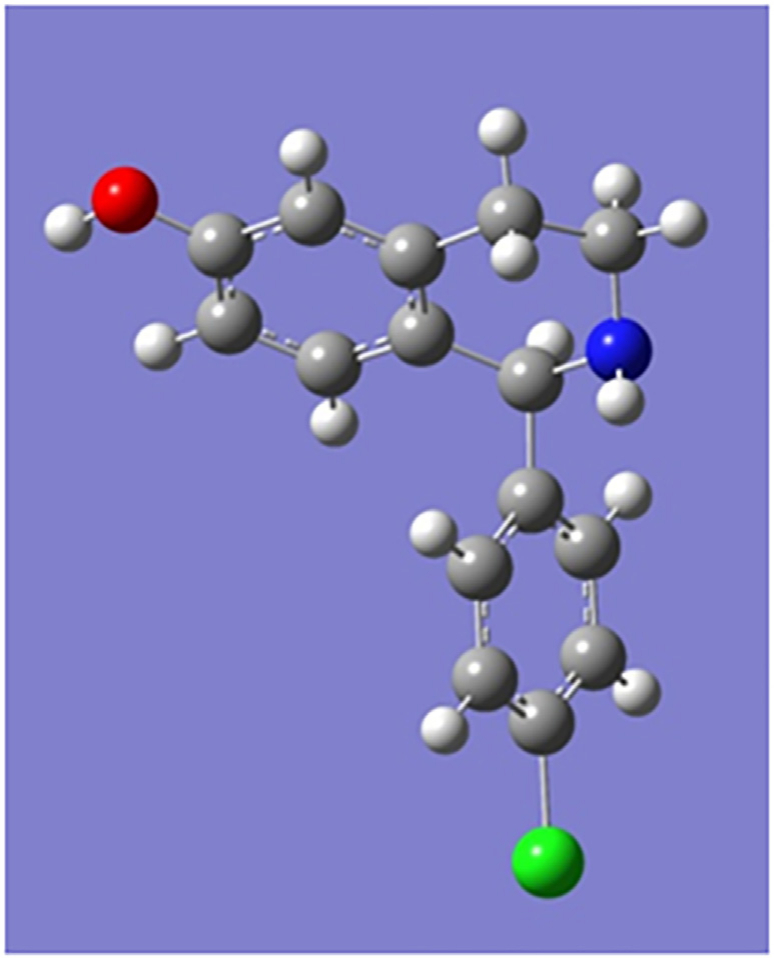
Geometry optimized structure of the most active 6-hydroxy-1,2,3,4-tetrahydroisoquinoline molecule (**1**).

Minimal topological difference for the *i*-th molecule,

(4) $$MTD_{i}=\underline{s}+\overset{M}{\underset{j=1}{\sum }}\;\varepsilon_{j}\cdot x_{ij};\;i=1,....,N,$$

where $\underline{s}$ represents the number of points common to all the 19 molecules in the hyperstructure (H), *j* stands for the vertices and varies from 1 to 20 in our case. A matrix was constructed for the binary components *x*_*ij*_, which can take the value of 1 or 0, depending on whether the vertex *j* is respectively occupied by an atom of molecule *i* or not. We therefore obtained a (19 × 20) matrix representing our 19 molecules and 20 vertices. The values of the entries of the matrix *ε*_*j*_ = could either be -1, 0 or +1 respectively representing the vertices supposed to belong to the cavity of the receptor (hence of benefit to biological activity), to the exterior of the receptor (not relevant for activity) and to the receptor walls (detrimental for activity) (Duda-Seiman et al., 2006). Figure 3 shows the superposition of the conformations of minimal energy. The hypermolecule that is formed by this superposition has 20 vertices and it is presented in Figure 4. Molecular modeling and *MTD* were performed with the geometry optimized structure of the 19 molecules using the MOE package (Chemical Computing Group Inc., 2010). The 19 antimalarial agents minimal energy conformations optimized with B3LYP (Becke, 1988; Clark et al., 1983; Curtiss et al., 1992; Ditchfield et al., 1971; Francl et al., 1982; Frisch et al., 1984; Hariharan and Pople, 1974; Hehre et al., 1972; Lee et al., 1988; Wiberg, 2004) of the DFT with the 6-311+G (d,p) basis set, were superposed on the most active molecule (**1**) of Table 1.

### 2.6 Other molecular descriptors

11 descriptors that best fit the regression model were selected by the QuaSAR-Contingency of MOE and 10 descriptors are described as follows:

#### 2.6.1 Hydrophobicity parameter

Hydrophobicity or lipophilicity is measured as the relative affinity of a molecule for a non-polar phase versus that for water. The partition coefficient of a molecule is very important for drug activity. The logarithm of the partition coefficient for *n*-octanol/water (log*P*) is a property that governs its partitioning into a non-aqueous phase (*n*-octanol). It was determined using the BROOD software provided by OpenEye (Advanced Chemistry Development, Inc., 2010). The smaller values of log*P* imply an increase in hydrophobicity of the solute.

#### 2.6.2 Molar refractivity

Molar refractivity is related to the polarizability of a substance. It characterizes the size of the substituents and the steric effect in intermolecular interactions. Molar refractivity terms are interpreted to reflect drug-receptor dispersion interaction. It is the additive and constitutive property of a compound which is easily and unambiguously measurable. This was computed using the ACD-Lab software (Advanced Chemistry Development, Inc., 2010), as defined in Eq. (5).

(5) $$MR=\frac{4}{3}\pi N\alpha ,$$

where *π* is the usual irrational real number = 3.1416, *N* is the Avogadro's number and *α* is polarizability.

#### 2.6.3 Potential energies and electrostatic energies and number of rotatable bonds

These descriptors were computed using the MOPAC module of the MOE package (CCG Inc., 2010) and represented as *E*_*p*_.

#### 2.6.4 Dipole moments

This was determined from the geometry optimized structures derived from Gaussian 09W (Gaussian, Inc., 2009a, 2009b).

#### 2.6.5 Chemical hardness

This was computed as the difference between the energies of the lowest unoccupied molecular orbital (LUMO) and the highest occupied molecular orbital (HOMO), as proposed by Parr, Yang and Pierson (Parr and Yang, 1989; Parr and Pearson, 1983), with the LUMO and HOMO energies (eq. (1)) derived from the geometry optimized structures.

#### 2.6.6 Electrophilicity

The global electrophilicity indices (*ω*) of the molecules, Eq. (2), which represent their electrophilic character, were defined from Eqs. (1) and (3) from the electronic chemical potential values (*μ*) and their chemical hardness (*η*).

The electrophilicity index value (*ω*) of each molecule was defined by Eqs. (2) and (3) as encompassing the propensity of the electrophilic molecule to acquire an additional electronic charge (i.e. the square of the electronic chemical potential (*μ*^2^)), which is actually the square of its electronegativity as well as its resistance of the molecular system to exchange electronic charge with its environment, as defined by *η* (Domingo et al., 2002; Lacerda et al., 2010).

#### 2.6.7 Two-dimensional polar surface area (TDPSA)

This was computed using BROOD software (OpenEye Scientific Software, Inc., 2006).

### 2.7 Fragment search and filtering for pharmacokinetic properties of suitable analogues

**Figure 5:**
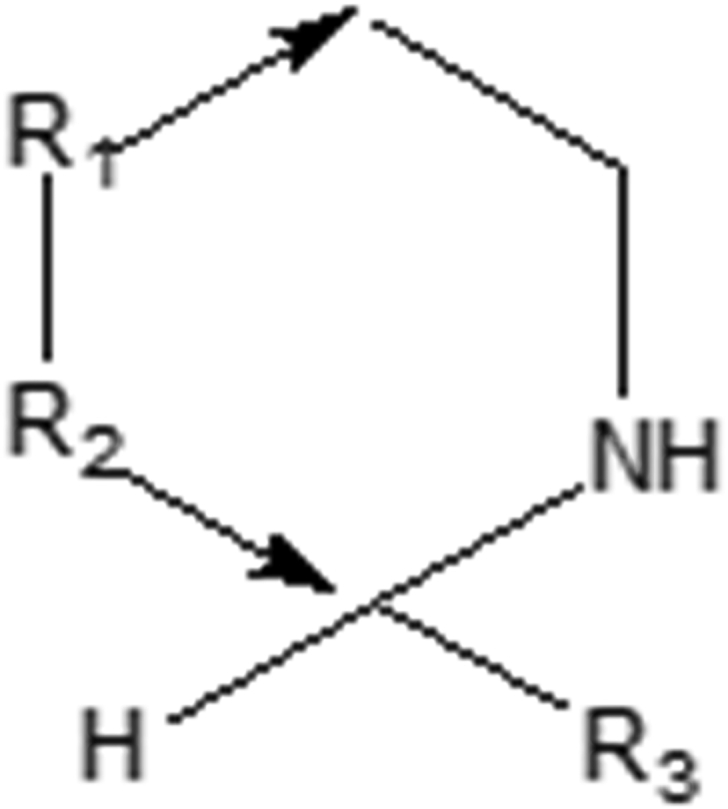
Scaffold structure and position of R-groups indicates.

In the design of antimalarial agents, we made use of the following core structure, as shown in Figure 5. Some suitable fragments for the bioisosteric replacement at positions R_1/2_ and R_3_ were selected using the MedChem Transformation module of MOE (Chemical Computing Group Inc., 2010). The proposed analogues were then designed and geometry optimized using the previously described procedure, molecular descriptors for the QSAR were then calculated and used to determine the predicted pIC_50_ values, while the pharmacokinetic properties were predicted using QikProp (Schrödinger, 2009). These proposed analogues were then used to design suitable ligands to be screened. An initial designed fragment search from a subset of aromatic and hydrophobic fragments was selected as suitable and diverse enough for replacements at the positions R_1/2_ and R_3_ according to Figure 5. This was used to design a combinatorial library using medicinal chemistry rules implemented in the CombiGen modules of the MOE package (CCG Inc., 2010), following a previously described methodology (Frecer et al., 2009). The analogues designed were further filtered for properties known to drug-like compounds (Lipinski's criteria for drug-likeness), i.e, molecular weight between 0 and 750 g/mol, *n*-octanol/water LogP between -2 and 6.0, number of donors and acceptors between 1 and 20 and number of rotatable bond between 1 and 15 from Qikprop (Schrödinger, 2009).

### 2.8 ADME prediction

Descriptors often used to predict the absorption, distribution, metabolism and excretion (elimination) of compounds were computed using the QikProp software by implementing methods developed by the Jorgensen group (Schrödinger, 2009). Pharmacokinetically-related parameters include log P, the aqueous solubility, the partition coefficient for crossing the brain/blood barrier, the cell permeability parameter (Caco-2), the binding affinity to human serum albumin, the number of likely primary metabolic reactions, etc. An overall drug-likeness parameter (referred to as #stars) was also computed. This connotes the number of computed properties derived from QikProp that deviate from the known range values for 95% of known drugs.

## 3 Results and discussion

### 3.1 QSAR equation

In order to develop a correlation between antimalarial activity and the computed molecular descriptors (physicochemical properties), the Hansch approach was employed. The 19 synthesized compounds were divided into two sets; a set of 15 compounds for training the models and a test set of 4 compounds for validation (Table 1). The computed molecular descriptors for the compounds in the training and test sets are represented in Tables 2 and 3. Sequential multiple linear regression (MLR) analysis was conducted on the training set compounds with the goal of establishing a correlation between physicochemical properties and experimental activities.

**Table 2:** Computed molecular descriptors for the training set, used to obtain the QSAR models.

#	*logP*	*MR*	*TDPSA*	*μ*_*D*_	*E*_*p*_	*MTD*	*η*	*ω*
1	3.0	73.39	32	1.8076	48.4335	45	0.0979	0.0889
2	3.6	78.20	32	2.9481	49.1185	29	0.0960	0.0977
3	3.0	73.39	32	2.2771	46.2053	45	0.0.987	0.0874
4	2.2	75.05	41	2.2664	57.1440	43	0.0997	0.0743
5	2.3	81.52	50	3.2212	78.5233	42	0.0998	0.0717
6	2.1	81.52	50	3.1697	71.9990	43	0.0936	0.0698
7	3.2	76.21	32	1.8277	46.5528	45	0.0977	0.0893
8	3.3	74.56	32	3.1778	50.7209	37	0.0929	0.1064
9	4.2	93.73	32	1.0667	70.5986	39	0.0912	0.0975
10	2.8	68.81	32	2.2028	45.4703	57	0.0981	0.0857
11	2.5	68.81	32	1.6401	45.0356	45	0.0978	0.0878
12	2.4	79.54	78	5.8340	64.7792	27	0.0685	0.2127
13	3.0	82.68	41	2.9018	60.0532	45	0.0960	0.0857
14	1.2	76.52	98	5.1969	58.9141	44	0.0666	0.2035
15	2.7	73.63	32	1.4431	46.8571	45	0.1000	0.0763

**Table 3:** Computed molecular descriptors for the test set, used to validate the QSAR model.

#	*logP*	*MR*	*TDPSA*	*μ*_*D*_	*E*_*p*_	*MTD*	*η*	*ω*
16	2.4	68.597	32	1.0989	46.5928	61	0.1002	0.0780
17	1.8	74.64	78	5.4562	59.7424	41	0.0664	0.2188
18	3.2	76.215	32	2.2863	46.7146	45	0.0982	0.0887
19	2.5	68.808	32	2.1578	44.9500	45	0.0992	0.0854

Several equations were obtained, with the best correlation coefficients being between 0.74 and 0.75 [Eqs. (6), (7), and (9)]. Only the QSAR model shown in Eq. (8) had an *R*^2^ value of less than 0.74. The squared correlation coefficients (*R*^2^) of the QSAR models Q1, Q2, Q3 and Q4 were very similar, only varying within narrow limits. Besides, all the root mean square error (*RMSE*) values were located between 0.213 and 0.221. A high correlation coefficient and low RMSE value both show that the QSAR models could be considered to be statistically valid. All the models were further validated by Fischer statistics. The statistical parameters of the 4 best QSAR models have been shown in Table 4 (Q1, Q2, Q3, and Q4).

**Table 4:** Statistical parameters of derived QSAR models.

QSAR Model	*R*^*2*^	*RMSE*	*SDEP*	*F*
Q1	0.7434	0.2142	0.2015	9.862
Q2	0.7460	0.2131	0.2131	10.778
Q3	0.7275	0.2207	0.2207	9.797
Q4	0.7434	0.2142	0.2142	10.631

The regression analysis correlating all the molecular descriptors with activity was obtained as follows, for the 4 best QSAR models derived:

Model 1

(6) p*IC*_*50*_^exp^ = – log_10_IC_50_ = 2.300 - (0.032 x MTD) – (0.025 x TDPSA) - (0.132 x log P) - (0.001 x E_p_), (*R*^*2*^ = 0.743, *RMSE* = 0.214)

Model 2

(7) p*IC*_*50*_^exp^ = – log_10_IC_50_ = -1.815 - (0.031 x MTD) + (0.012 x TDPSA) - (14.812 x η) - (0.008 x E_p_), (*R*^*2*^ = 0.746, *RMSE* = 0.213)

Model 3

(8) p*IC*_*50*_^exp^ = – log_10_IC_50_ = 1.966 - (0.033 x MTD) – (0.139 x log P) - (13.652 x ω) - (0.013 x E_p_), (*R*^*2*^ = 0.728, *RMSE* = 0.221)

Model 4

(9) p*IC*_*50*_^exp^ = – log_10_IC_50_ = 2.283 - (0.032 x MTD) – (0.132 x log P) + (0.003 x η) - (0.025 x TDPSA), (*R*^*2*^ = 0.743, *RMSE* = 0.214)

**Figure 6:**
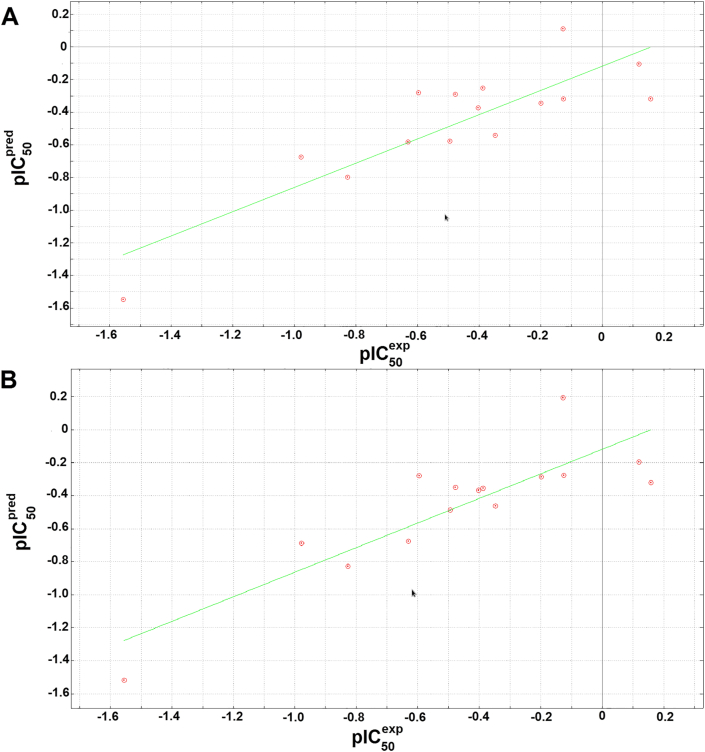
Cross-validated correlation plots for QSAR models for (A) model 1, and (B) model 2.

**Figure 7:**
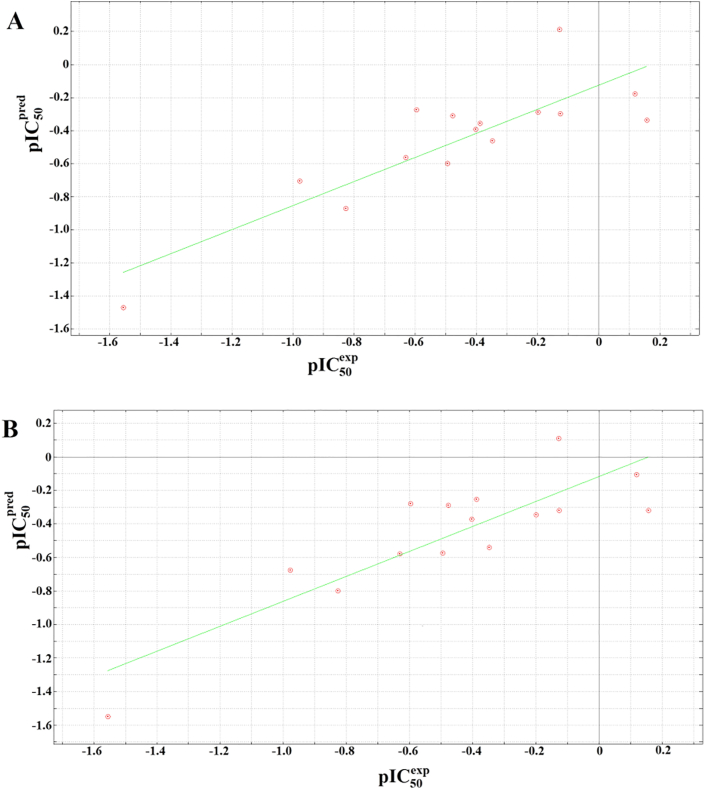
Cross-validated correlation plots for QSAR models for (A) model 3, and (B) model 4.

The cross-validated correlation plots for models Q1 and Q2 have been shown in Figure 6, while those for models Q3 and Q4 have been shown in Figure 7.

The regression relations include the minimal topological differences (MTD) of each molecules, which is a representation of the steric factor, as well as polarity (represented by the TDPSA parameters), the lipophilicity (represented by the computed logP values), the total potential energies from the forcefield, (which includes the electrostatic energies and changes due to number of rotatable bonds), the chemical hardness (η), and the electrophilicity (ω). The parameters not included in the best models included dipole moments, number of rotatable bonds and molar refractivity. Each model was internally validated by its ability to predict the activities (experimental pIC_50_s) by using the leave-one-out (LOO) method, leading to cross-validated correlation coefficients that could explain about three quarters (~75%) of the observed activities. Besides, each model showed low standard deviation of error prediction (SDEP) values (<0.22) and low root mean square error (*RMSE*) values (<0.22). *F*-statistics was used to access the statistical significance of each QSAR equation. Eq. (10) was used to determine the *F*-test:

(10) $$F_{p2-p1,n-p2}=\frac{SS_{mean}-SS_{pred}}{SS_{mean}}\times \frac{n-p2}{p2-p1}$$

where *SS*_*mean*_ is the sum of squares of the residuals or the differences between the (measured) experimental activities and their mean value, *SS*_*pred*_ is the sum of squares of the differences between the experimentally measured activities and their respective predicted activities, *n* is the number of compounds in the training set (15), while *p1* and *p2* are the respective number of parameters in the derived reference equations, i.e. Eqs. (6), (7), (8), and (9), i.e. for each of these equations, *p1* = 4 and *p2* = 5. In this scenario, all *F*-values are referred to as *F*_*5-4,15-5*_, i.e. *F*_*1,10*_ and have been shown in Table 4, all ~10, indicating that the probability level of the significant correlations lie between 0.9 and 0.95.

It must be also mentioned that the regression coefficients of the *MTD* parameter are negative in all derived models, implying that bulkiness (the steric factor) rather plays a negative role in binding towards the putative receptor. In designing more active analogues to this compound series, we had to be careful to avoid bulky groups and very polar groups (since the regression coefficients for TDPSA are also negative for models Q1 and Q4, corresponding to Eqs. (6) and (9)). This is also verified by the fact that the least active training set compounds (i.e. compounds **12** and **14**), having IC_50_ values of 6.727 and 35.978 μM, respectively, had the highest computed TDPSA values (78 and 98, respectively).

Each model was validated against the 4 compounds included in the validation set (not initially used to derive the models Q1 to Q4), showing the abilities of the models to predict the experimentally verified activities. This was represented by residuals (differences between experimental and predicted activities (Table 5)).

**Table 5:** Validation of derived QSAR models using residuals.

Validation Set	$\text{Residual }\left(pIC_{50}^{exp}-pIC_{50}^{pred}\right)$				RMSE			
	Q1	Q2	Q3	Q4	Q1	Q2	Q3	Q4
16	0.4043	0.4433	0.4940	0.3860	0.2870	0.1891	0.2131	0.1134
17	1.0077	1.2516	1.2906	1.0546	0.2234	0.2148	0.2365	0.1248
18	-0.2339	-0.2801	-0.2649	-0.2045	0.1145	0.1236	0.2135	0.2341
19	-0.5693	-0.5030	-0.4768	-0.5621	0.1478	0.2897	0.1278	0.2358

It was observed that the residual values showed that all four models were able to accurately predict the experimental pIC_50_ values (with residuals less than 0.5), except for compound **17**. It must be noted that compound 17 is the only nitro-containing compound in the test set (we also note that there are only two nitro-containing compounds within the training set, when compared with the other chemotypes like fluro, chloro, bromo and methoxy groups). Besides, the nitro compounds in the training set represents the weakest activities, meaning that their contribution towards the QSAR models could be insignificant. This observation could either be explained by the fact that the parameters used to derive models Q1 to Q4 were not accurately computed for the nitro compound or that the nitro compound simply did not fall within the domain of applicability of the derived QSAR models (Q1 to Q4).

### 3.2 Novel analogues

#### 3.2.1 Fragments for library design

**Figure 8:**
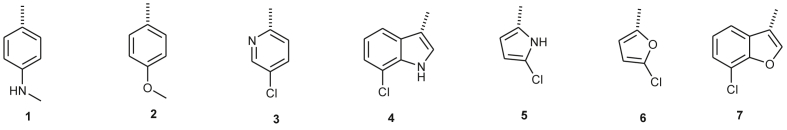
R_3_ fragments used in the design of library of anti-malarial agents.

**Figure 9:**
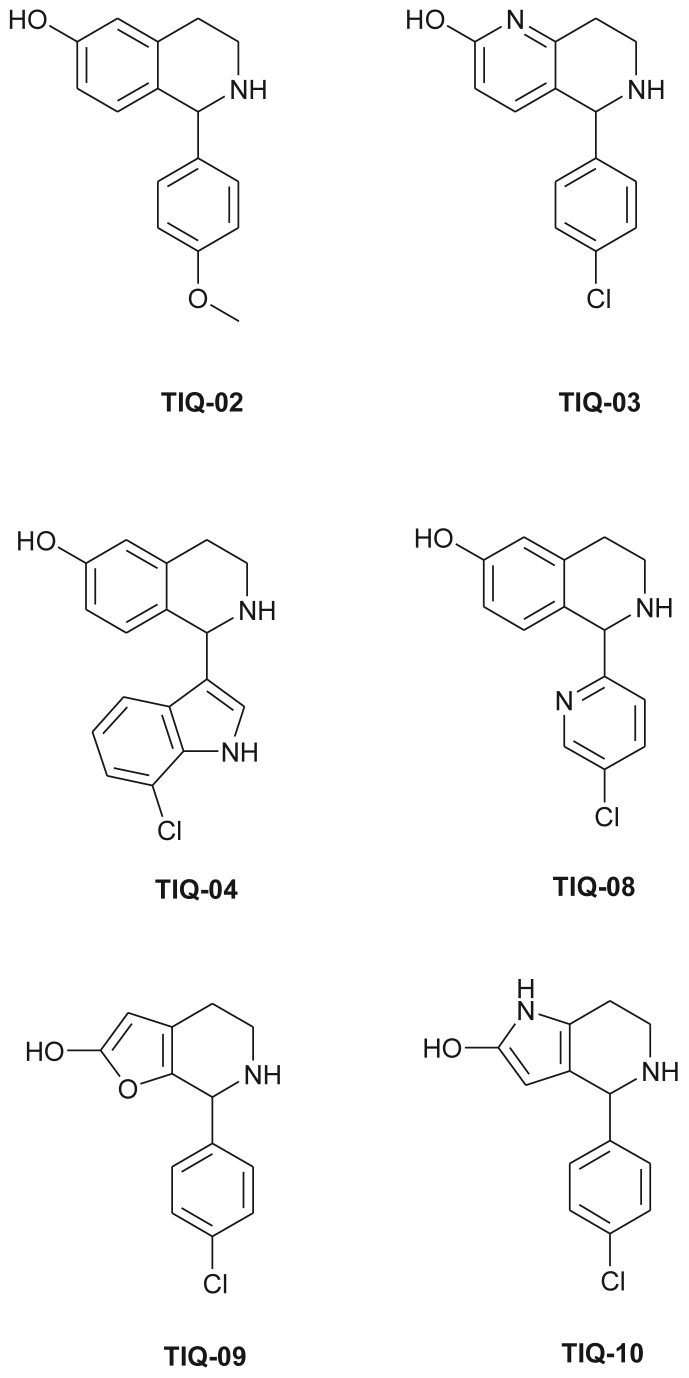
Chemical structures of predicted activities of six theoretically most potent analogues of 6-hydroxy-1,2,3,4-tetrahydrosioquinoline designed against *P. falciparum*.

These consisted of 7 R_3_ fragments presented in Figure 8. Other positions of the scaffold, apart from position R_3_ were also modified to improve on the activity. The compounds obtained include those shown in Figure 9.

#### 3.2.2 In silico search

The best designed analogues of 6-hydroxyl-1,2,3,4-tetrahydroisoquinoline were screened *in silico* by using the calculated molecular descriptors used to derive the four QSAR Eqs. (6), (7), (8), and (9). The predicted antimalarial activities of the analogues designed within this study were then computed using the specific QSAR equations (Q1 to Q4), shown in Eqs. (6), (7), (8), and (9). The analogues were then ranked according to the predicted antimalarial activities according to the estimated pIC_50_ values. The analogues showing the highest predicted activities (referred to as virtual hits) have been shown in Table 6. The design analogue with the most promising antimalarial activity, **TIQ-08**, contains two aromatic rings, one heterocyclic aromatic ring (R_3_-group) attached to the heterocyclic cyclohexane ring that fills the hydrophobic and aromatic pocket occupied by the 4-chlorophenyl in the most active compound in the training set. This compound was predicted to have an IC_50_ value of 1.175 μM according to the QSAR Model 2.

**Table 6:** Molecular weights (MW) and computed QSAR descriptors and predicted activities of six theoretically most potent analogs of 6-hydroxy-1,2,3,4-tetrahydrosioquinoline designed against *P. falciparum*.

Analog	LogP^a^	MW^b^	NRB^c^	MTD	TDPSA^c^	E_p_	η	*ω*	$pIC_{50}^{pred}$ (Model 1)	$pIC_{50}^{pred}$ (Model 2)	$pIC_{50}^{pred}$ (Model 3)	$pIC_{50}^{pred}$ (Model 4)	$IC_{50}^{pred}$ (μM, Model 1)	$IC_{50}^{pred}$ (μM, Model 2)	$IC_{50}^{pred}$ (μM, Model 3)	$IC_{50}^{pred}$ (μM, Model 4)
TIQ-02	2.2	255.31	2	43	41	55.064	0.827	0.068	-0.446	-15.341	-1.400	-0.406	2.796	2.196	25.132	2.546
TIQ-03	2.1	260.72	1	45	45	34.274	0.971	0.459	-0.576	-17.321	-6.520	-0.556	3.771	2.092	33.113	3.600
TIQ-04	3.1	298.77	1	37	48	45.084	0.372	0.152	-0.538	-8.255	-2.346	-0.509	3.454	1.800	221.685	3.229
TIQ-08	1.7	260.72	1	45	45	75.038	0.594	0.418	-0.564	-12.070	-6.439	-0.505	3.668	1.175	27.459	3.196
TIQ-09	2.3	249.69	1	43	45	92.87	0.547	0.068	-0.597	-11.458	-1.914	-0.520	3.958	2.868	82.004	3.311
TIQ-10	2.1	248.71	1	43	48	38.495	0.408	0.170	-0.592	-8.928	-2.565	-0.569	3.906	8.466	367.120	3.707

a P is defined as the *n*-octanol/water partition coefficient.

b MW is the molecular weight of the compound.

c NRB is the number of rotatable bonds and TDPSA is the two dimensional polarity surface area (logp P, MW, NRB, 2DPSA were calculated using BROOD [12]).

#### 3.2.3 ADME-related properties and hit prioritization

Incorporation of ADME properties into lead selection is very important in order to determine the pharmacokinetic properties of the selected virtual hits. Some pharmacokinetically-relevant molecular properties computed for the TIQ-analogues were used to prioritize the choice of virtual hits for further development. Some 13 of these descriptors have been shown in Table 7 (the chemical structures are shown in Figure 9). The overall ADME-compliance is reflected by the low values of #stars. the parameter that indicates the number of properties falling out of the optimal range of values for 95% of known drugs. The six designed analogues with the best computed antimalarial activities and pharmacokinetic profiles have been shown, with only one compound (**TIQ-10,** Figure 9) violating the optimum range of the ADME-related properties in one descriptor. We expect that some of these designed analogues could further be developed.

**Table 7:** Computed ADMET-related parameters for newly designed analogs.

Hits	#stars	MW	SASA	FOSA	Volume	NRB	HB_don_	HB_acc_	logP_o/w_	logS_wat_	logK_hsa_	logBB	BIP_caco_	#metab
TIQ-02	0	255.32	501.228	197.373	858.666	2	2	3	2.423	-2.642	0.21	0.172	466.401	4
TIQ-03	0	260.72	488.775	110.363	821.025	1	2	2.5	2.512	-3.019	0.234	0.258	327.856	4
TIQ-04	0	298.77	523.246	108.051	903.608	1	3	2.25	2.892	-3.412	0.365	0.216	306.337	3
TIQ-08	0	260.72	489.188	108.839	822.53	1	2	3.25	2.315	-2.853	0.132	0.324	384.408	4
TIQ-09	0	249.69	466.843	117.788	775.681	1	2	2.000	2.497	-2.748	0.185	0.405	458.461	4
TIQ-10	1	248.71	473.853	116.618	785.775	1	3	1.5∗	2.285	-2.684	0.139	0.172	263.105	3

MW: molecular weight in Da (range for 95% of drug: 130-725Da).

SASA: total solvent-accessible molecular surface, in Hydrophobic portion of the solvent-accessible molecular surface, in Å^2^ (range for 95% of drug: 300-750Å^2^).

Volume: total volume of molecule enclose by solvent-accessible molecular surface, in Å^3^.

NRB: number of rotatable bonds (range for 95% of drug: 0–15).

HB_don_: number of hydrogen bonds donated by the molecule (range for 95% of drug: 0–6).

HB_acc_: number of hydrogen bonds accepted by the molecule (range for 95% of drug: 2–20).

LogP_o/w_: logarithm of partition coefficient between n-octanol and water phases (range for 95% of drug: -2 to 6).

LogS_wat_: logarithm of aqueous solubility (range for 95% of drug: -6.0 to 0.5).

LogKhsa: logarithm of predicted binding constant to human serum albumin (range for 95% of drug: -1.5 to 1.2).

LogBB: logarithm of predicted blood/brain barrier partition coefficient (range for 95% of drug: -3.0 to 1.0).

BIPcaco: predicted apparent caco-2 cell membrane permeability in Boehringer-ingelheim scale in nm/s(range for 95% of drug: <5 low, >100 high).

#metab: number of likely metabolic reactions.

## 4 Conclusions

This study involved the use of computer models to generate a library of small molecules based on the hydroxyl-1,2,3,4-tetrahydroisoquinoline core, which includes compounds with predicted antimalarial activities. The designed analogues with the predicted activities show IC_50_ values within the vicinity of the most active compounds in the training set, but with favourable predicted pharmacokinetic parameters. The most promising designed analogues were shown to be drug-like molecules and have predicted favourable ADMET profiles. The importance of the study is highlighted by the fact that the proposed analogues could help chemists interested in preparing novel THIQs with the potential to be developed into next generation antimalarials.

## Declarations

### Author contribution statement

Joelle Ngo Hanna, Vincent de Paul N. Nziko: Performed the experiments; Wrote the paper.

Fidele Ntie-Kang: Conceived and designed the experiments; Analyzed and interpreted the data; Contributed reagents, materials, analysis tools or data; Wrote the paper.

James A. Mbah, Flavien A. A. Toze: Conceived and designed the experiments; Analyzed and interpreted the data; Contributed reagents, materials, analysis tools or data.

### Funding statement

F.N.K. was supported by the German Academic Exchange Services (10.13039/501100001655DAAD) for a guest professorship at TU Dresden.

### Data availability statement

Data included in article/supplementary material/referenced in article.

### Declaration of interests statement

The authors declare no conflict of interest.

### Additional information

No additional information is available for this paper.
